# 3D stereophotogrammetry in children and adolescents with Scleroderma En Coup De Sabre/Parry‐Romberg Syndrome: Description of a novel method for monitoring disease progression

**DOI:** 10.1002/ski2.132

**Published:** 2022-07-05

**Authors:** Rutger ter Horst, Thomas J. J. Maal, Martien J. J. de Koning, Jorre S. Mertens, Ellen J. H. Schatorjé, Esther P. Hoppenreijs, Marieke M. B. Seyger

**Affiliations:** ^1^ Department of Oral and Maxillofacial Surgery Radboud University Medical Center Nijmegen The Netherlands; ^2^ Department of Dermatology Radboud University Medical Center Nijmegen The Netherlands; ^3^ Department of Pediatrics Pediatric Rheumatology, Radboud University Medical Center Nijmegen The Netherlands

## Abstract

**Background:**

The diagnosis of Scleroderma En Coup de Sabre (ECDS)/Parry Romberg Syndrome (PRS) is mainly based on characteristic clinical findings. Methods to objectively monitor the course of the disease in a standardized way are lacking.

**Objectives:**

This descriptive, retrospective, single centre cohort study aims to describe the contribution of 3D photographs in the assessment of the degree of facial asymmetry changes over time in growing children and adolescents with ECDS and PRS.

**Methods:**

Six patients diagnosed with ECDS/PRS, with a follow‐up period of at least 24 months and at least three 3D photographs were included. Mirroring these 3D photographs was automatically performed using surface‐based matching to generate a colour‐coded distance map, illustrating the inter‐surface distance and thereby asymmetry between the original and mirrored 3D photographs. The percentage of absolute distances between the original and mirrored 3D photograph were calculated.

**Results:**

In two patients, impressive decreases in the percentages of absolute distance levels over time were found, whereas the other patients did not show progression of asymmetry over time.

**Conclusion:**

This study shows the potential of 3D stereophotogrammetry as an objective tool to measure disease activity over time in patients with ECDS/PRS.

## Graphical abstract

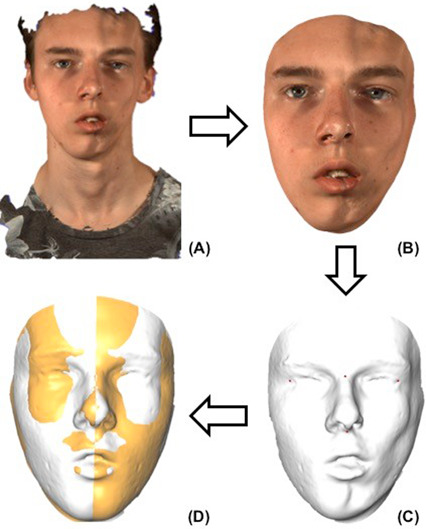

1

**What is already known about this topic?**

- Currently, the diagnosis of ECDS/PRS is mainly based on characteristic clinical findings. To the best of our knowledge, methods to objectively monitor the course of the disease in a standardized way are lacking.

**What does this study add?**

- This study shows the potential of 3D stereophotogrammetry as an objective tool to measure disease activity over time in patients with ECDS/PRS.

## 2 INTRODUCTION

Morphea is an idiopathic sclerosing disorder of the skin and underlying tissues. A subtype of morphea, Scleroderma En Coup De Sabre (ECDS), affects the face and/or scalp with a linear induration of the skin and sometimes involvement of the underlying muscle and bone. Parry‐Romberg Syndrome (PRS), also known as Progressive Hemifacial Atrophy, presents as a loss of tissue on one side of the face. It may involve dermis, subcutaneous tissue, muscles and underlying bone.^1^, ^2^, ^3^ It is still controversial whether PRS is a variant of facial linear scleroderma or an independent disorder. Recent studies, however, have concluded that both diseases are on the same spectrum of disease, possibly sharing a similar pathogenesis.^4^ Therefore, some authors prefer to use the term linear scleroderma of the face to cover both ends of the spectrum.^1^

When children and adolescents are affected by ECDS and/or PRS, the deformities potentially have influence on their facial attractiveness and psychological wellbeing.^5^, ^6^ Currently, the diagnosis of ECDS/PRS is mainly based on characteristic clinical findings. The management of linear scleroderma of the face is challenging. Recently, minimum standards of care for children and adolescents with localized scleroderma (including scleroderma of the face) have been proposed by members of the Paediatric Rheumatology European Society Scleroderma Working Group.^7^ In their recommendations, the aim of treatment is to reach disease inactivity, defined as no erythema, no new lesions over the past 3 months, no worsening of skin thickness, no worsening of joint contractures, physician global assessment on visual analogue scale of 0 and no active extracutaneous involvement. The authors state that inactivity in deeper lesions can be harder to define by these parameters. Although erythema, induration and superficial extension of lesions can be assessed in ECDS and PRS, the deeper extension or progression of hemifacial atrophy is very difficult to monitor. Especially in growing children and adolescents who naturally show craniofacial growth,^8^, ^9^ monitoring the disease is even more challenging. To the best of our knowledge, methods to objectively monitor the course of the disease in a standardized way are lacking.

To capture a facial soft tissue surface, three‐dimensional (3D) stereophotogrammetry has gained great interest over the past decade in the field of Oral and Maxillofacial Surgery (OMFS). In patients who undergo maxillofacial orthognathic surgery, for example, 3D stereophotogrammetry is widely used to evaluate preoperative and postoperative three‐dimensional changes of the face. The extra dimension of a 3D photograph makes it possible to calculate even the smallest surface differences. In addition, using surface‐based registration techniques 3D photographs acquired at different stages of the disease and during treatment can be matched.^10^, ^11^ This accurate quantification of the facial soft tissue surface is a validated method and can therefore be used to evaluate the progression of facial growth disorders over time.^12^, ^13^ In case of disease activity of ECDS/PRS, deeper extension of skin lesions and/or progression of hemifacial atrophy is expected. We hypothesize that 3D photographs are able to accurately measure changes in facial asymmetry in ECDS/PRS and could therefore serve as a tool to monitor disease activity.

This study aims to describe the contribution of 3D photographs in the assessment of changes in facial asymmetry over time in growing children and adolescents with ECDS and PRS.

## 3 MATERIAL AND METHODS

### 3.1 Patient selection

In this descriptive, retrospective, single centre cohort study, patients diagnosed with ECDS/PRS treated at the Department of Paediatric Rheumatology and Dermatology at the Radboud University Medical Centre were selected. A few years ago, we decided to start making 3D photographs of all patients with ECDS/PRS visiting our combined outpatient clinic meaning that at the moment of the first 3D photograph, some patients already completed their systemic treatment while others were still on active drug or just commenced their systemic treatment. For this retrospective analysis, patients were included if they met the following inclusion criteria: (i) age at onset of ECDS/PRS <18 years, (ii) availability of at least three 3D photographs with an interval of 6 months during a follow‐up period of at least 24 months. Patients who had (orthognathic) facial surgery during follow‐up were not included.

This study was conducted in accordance with the World Medical Association Declaration of Helsinki on medical research ethics. The study was approved by the institutional medical ethical authority (file number W13_303 # 13.17.373) and informed consent was acquired for all patients who were enrolled in the study. All image data were anonymized and de‐identified prior to analysis.

### 3.2 Patient characteristics

The following items were retrospectively collected from medical electronic charts: age at onset of disease, age at first 3D photograph, age at inclusion in this study, sex, affected side and region of the face and previous and/or current medical treatment.

### 3.3 Image acquisition

3D photographs were acquired using a stereophotogrammetrical camera set‐up (3dMD face™ System, 3dMD LCC, Atlanta, GA, USA). All photographs were taken using a standardized protocol^10^: Patients were positioned in a natural head position with their eyes open, the forehead free of facial hair and their facial musculature in a relaxed state with loosely closed lips, in order to avoid overestimation of facial asymmetry.

### 3.4 Analysis of facial asymmetry

**FIGURE 1:**
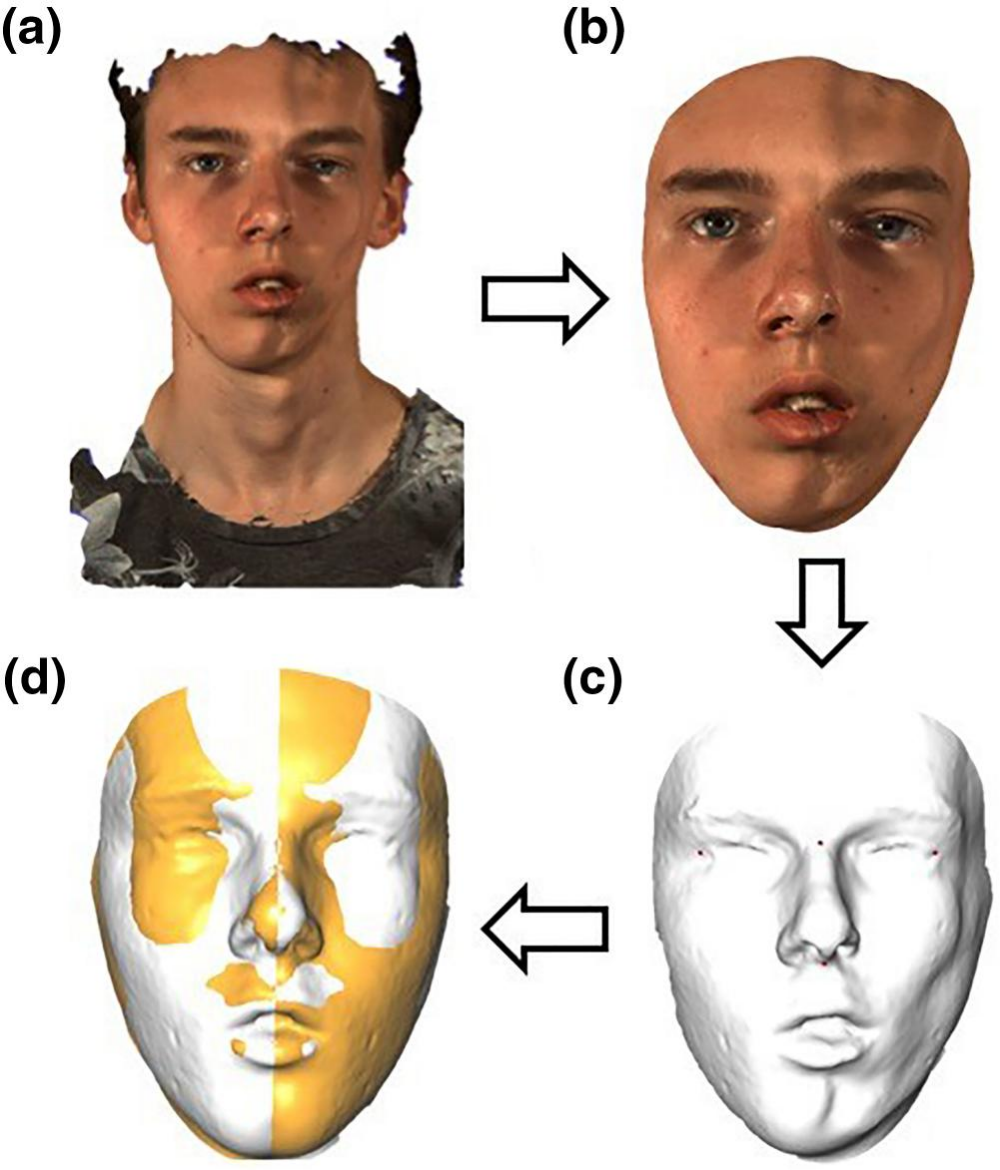
Alignment of the 3D photographs

**FIGURE 2:**
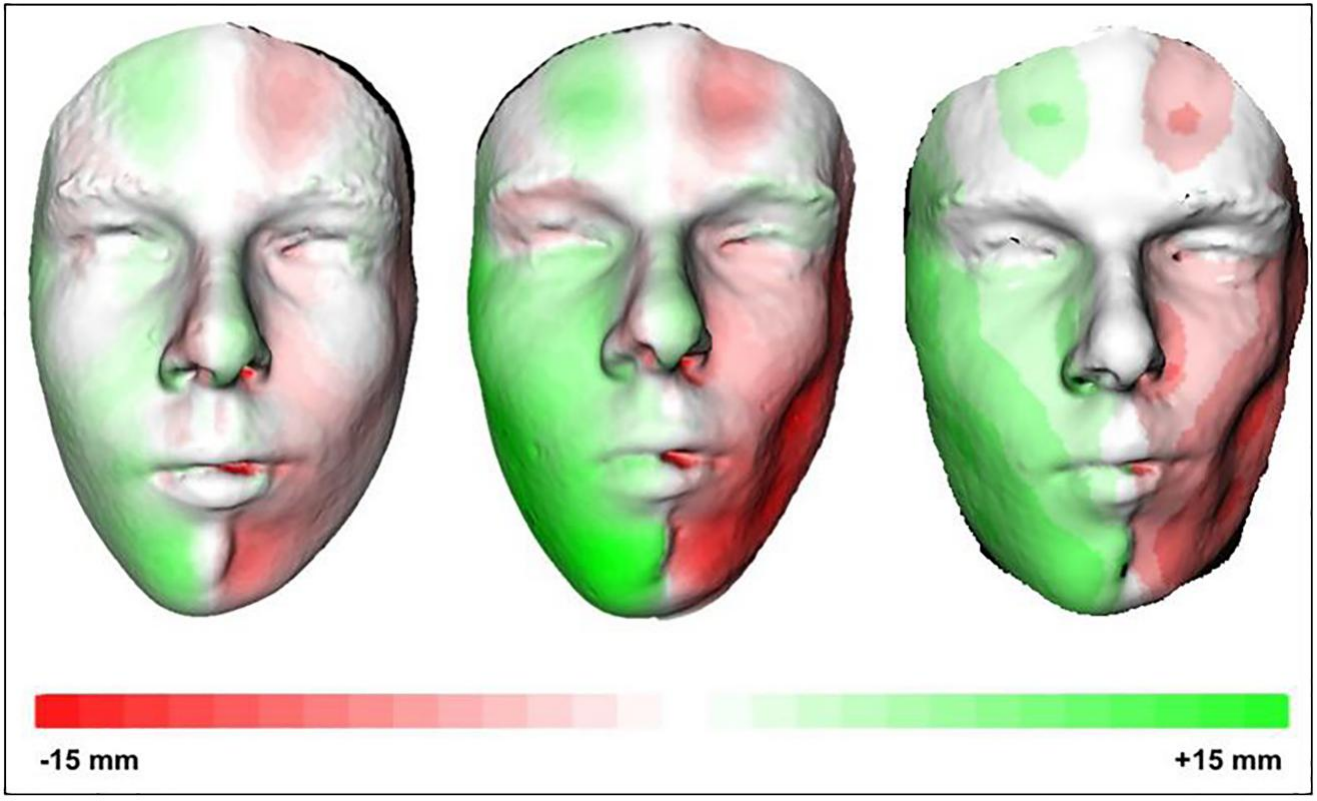
A 3D coloured histogram (distance map) of patient 6 over time. The green color indicates a volume increase and the red color a volume loss

To analyze facial asymmetry, all 3D photographs were processed as described by Verhoeven et al.^13^ In summary, four consecutive steps were needed to achieve a fine alignment of the 3D photographs (Figure 1). First, the region of interest was selected and confounding areas such as the neck were excluded. Second, a mirrored 3D facial surface (mirrored 3D photograph) was created using the midsagittal facial plane. For this step, an absolute perfect 3D photograph was necessary. Since our patients were in a growing phase, comparing 3D photographs from different time points was not possible. By creating a mirrored 3D photograph, patients were their own control group and a bias due to normal growth was excluded. After finishing this step an original and a created (mirrored) 3D photograph were available for detailed asymmetry analysis. Third, both original and mirrored 3D photographs were roughly aligned. After initial alignment, accurate surface‐based alignment was automatically performed using surface‐based matching (Iterative Closest Point algorithm). Last, a colour‐coded distance map was generated to illustrate the inter‐surface distance between the original and mirrored 3D photographs (Figure 2). Since there is great discussion of the mean facial asymmetry seen in the healthy paediatric and adolescent population, the obtained values were not corrected for this.^14^, ^15^

### 3.5 Analysis of facial asymmetry over time

To analyze facial asymmetry over time for every individual, at least three colour‐coded distance maps were created for the 3D photographs acquired at different timepoints using the abovementioned method. All patients included in the study had a follow‐up period of at least 24 months, which made it possible to analyze potential progression of facial asymmetry.

### 3.6 Statistical analysis

From the computed colour‐coded distance maps the absolute mean value and standard deviations were calculated using MATLAB version R2019b (The Mathworks, Inc, Natick, Massachusetts, USA) for all patients and different timepoints. Descriptive analysis was performed using IBM SPSS Statistics, Version 25 (IBM Corp., Armonk, NY, USA).

## 4 RESULTS

### 4.1 Patient characteristics

In this descriptive pilot study six patients (50% females) were included (Table 1) with a median age at inclusion of 13 years [range 9–19 years] and a median duration of their disease of 5.5 years [range 5–8 years]. The median follow‐up time from start of first 3D photograph to moment of analysis was 32 months [range 24–42 months].

**TABLE 1:** Patient characteristics

	Age at inclusion	Sex	Age of onset disease	Age at first 3D	Diagnose	Localization	Affected side	Treatment	Start treatment before first 3D photograph	Still on medication at moment of analysis
Patient 1^a^	11	F	5	8	ECDS	Forehead/chin	Left	MTX	Yes	Yes
Patient 2^b^	19	M	11	16	ECDS/PRS	Forehead/chin	Left	MTX	Yes	No
Patient 3^c^	16	F	11	13	ECDS/PRS	Mandible/Chin	Left	MTX/MMF	Yes	No
Patient 4^d^	11	F	3	9	ECDS/PRS	Upper lip/eye	Left	MTX	Yes	Yes
Patient 5^e^	14	M	9	12	ECDS	Forehead	Left	MTX	No	Yes
Patient 6^a^	9	M	4	7	ECDS/PRS	Mandible/chin	Left	MTX	Yes	Yes

Abbreviations: ECDS, Scleroderma En Coup de Sabre; F, Female; M, Male; MMF, Mycophenolate Mofetil; MTX, Methotrexate; PRS, Parry‐Romberg Syndrome.

^a^ Patient 1 and 6 started MTX 2 years prior to the first 3D photograph, and are still on MTX for 24 and 42 months, respectively.

^b^ Patient 2 received MTX for a period of 18 months, 15 months prior to the first 3D photograph. No medication during follow‐up.

^c^ Patient 3 received MTX for a period of 1 year and MMF for a period of 15 months prior to the first 3D photograph. No medication during follow‐up.

^d^ Patient 4 started MTX 2 years prior to the first 3D photograph, and is still on MTX for 24 months.

^e^ Patient 5 received no medical treatment prior to the first 3D photograph, and is on MTX since first 3D photograph (24 months).

Prior to the first 3D photograph, 5 out of 6 patients (83%) were treated with methotrexate (MTX). Of these 5 patients, 4 received this treatment for a period of at least 18 months [range 18–24 months] and one was treated with MTX for 12 months followed by mycophenolate mofetil (MMF) for a period of 15 months. Only one patient had the first 3D photograph taken before commencement of systemic therapy (patient 5). Apart from patient 2 and 3, all patients were still on MTX treatment when analysing the results.

### 4.2 Soft tissue asymmetry

Table 2 presents the absolute 3D distance measurements of the mirrored 3D photograph compared to the original 3D photograph.

**TABLE 2:** Absolute differences for the entire face using three‐dimensional distance maps, per patient

	Follow‐up period^a^	*T*_mean (mm)_	*T*_≤1 mm (%)_	*T*_≤2 mm (%)_	*T*_≤5 mm (%)_
Patient 1	0	1.8	50	65	92
	6	1.7	39	63	97
	18	2.1	43	60	89
	30	1.7	45	66	95
	36	1.7	39	65	99
	42	1.8	43	64	94
Patient 2	0	2.0	42	64	92
	6	1.8	42	61	95
	18	2.7	26	41	97
	24	3.2	21	39	75
	30	5.9	16	25	47
	36	6.4	14	22	44
Patient 3	0	1.2	54	79	99
	24	3.5	23	37	67
	36	3.5	23	36	69
Patient 4	0	0.7	74	95	100
	12	0.7	73	92	100
	21	0.7	74	94	100
	27	0.7	72	94	100
Patient 5	0	1.9	40	61	92
	9	1.9	39	58	96
	24	1.2	56	79	99
Patient 6	0	0.4	90	97	100
	9	1.3	60	73	98
	24	0.6	85	96	100

Abbreviations: *T*_mean_, mean absolute difference (Euclidean distance) in millimetres; *T*_≤1 mm (%)_, percentage of simulations within 1‐mm absolute minimal level of difference; *T*_≤2 mm (%)_, percentage of simulations within 2‐mm absolute minimal level of difference; *T*_≤5 mm (%)_, percentage of simulations within 5‐mm absolute minimal level of difference.

^a^ Retrospective follow‐up period in months, whereby 0 is defined as moment of first 3D photograph.

The percentage of absolute distances between the original and mirrored 3D photograph were calculated for cut‐off values of 1 mm, 2 and 5 mm. These values describe which percentage of the face (3D photograph) lies within this range, with 100 indicating that all and 0 indicating that no surface points lay within the given range, respectively. In patient 2, for example, 36 months after baseline, only 44% of skin surface of the face lies within the cut‐off value of 5 mm, compared to 92% at baseline. This indicates an absolute increase in asymmetry over time. The most impressive decreases in the percentages of absolute distance level less or equal in all three cut‐off groups over time were noted in patients 2 and 3. Remarkably, only these two patients did not receive active drug therapy during the period 3D photographs were taken. No clear difference in progression of asymmetry over time could be identified for the other patients.

## 5 DISCUSSION

Linear scleroderma of the face is a rare condition mainly affecting children and adolescents. Since monitoring progression of hemifacial atrophy over time in an individual who naturally shows craniofacial growth is exceptionally challenging, the decision to quit or adjust medical treatment is mainly based on subjective parameters and expert opinions. To the best of our knowledge, a non‐invasive method to objectively monitor the course ECDS and PRS over time in a standardized way is lacking. This study is the first to report that 3D photography can be used to objectively monitor disease progression over time in a growing individual.

3D stereophotogrammetry is known to be a safe and non‐invasive imaging modality. It is a validated method widely used in OMFS which captures high quality 3D photographs of the soft tissue facial profile in less than 2 milliseconds without using radiation.^16^ Here we present a retrospective case series in which progression of facial asymmetry over time was found in two patients. Prior to their first 3D photograph, one patient (patient 2) was treated with MTX for 18 months, while another patient (patient 3) used MTX for 12 months and switched to mycophenolate mofetil due to gastro‐intestinal complaints. Both patients refused further systemic treatment as they were satisfied with the clinical result and were reluctant to prolongate their treatment episode. The other four patients were still on MTX treatment at the moment of analysis (with a median treatment duration of 18 months) and showed no explicit progression of the facial asymmetry over time. These results might suggest a favourable effect of prolonged MTX treatment on the prevention of asymmetry. However, given the relatively small number of included patients, the descriptive nature of this study, lack of correction for confounding factors, solid conclusions on the efficacy of MTX on the prevention of facial asymmetry cannot be drawn.

As noted before, children and adolescents with facial asymmetry may suffer from negative psychological consequences. The degree of asymmetry causing these problems, however, is certainly not a predefined absolute value. In addition, every face has a slight degree of asymmetry. *Cho et al*. quantified a standardized normal craniofacial form and a baseline craniofacial asymmetry.^14^ They found a mean facial asymmetry in patients 0–18 years of age of 1.2 ± 0.6 mm. There was no statistical difference between age, sex and race. These given values, however, are significantly higher in comparison with the values published by Kuijpers et al.,^15^ who found an absolute mean facial asymmetry of 0.483 ± 0.148 mm. Since there is no consensus on the mean facial asymmetry of healthy children and adolescents, our results were not corrected for this. However, as the percentage of absolute distances between the original and mirrored 3D photograph in this study were calculated for cut‐off values of 1, 2 and 5 mm, the percentages of 2 and 5 mm are absolutely indicative for a non‐physiological facial asymmetry. A large prospective study is needed for this specific patient population to elucidate if this information can be used to adjust medical treatment and make clinical decision based on the progression measured using 3D stereophotogrammetry.

For clinicians, diagnosing severe facial asymmetry is not a challenge. The additional value of 3D photography is mainly in those patients showing mild progression of asymmetry over time. An overall progression of facial asymmetry of 1–2 mm between every consultation, for example, is very difficult to monitor. When using 3D photographs and the abovementioned method to analyze facial asymmetry, the clinician is able to compare the current situation with the last check‐up. With a system error of 0.2 mm^17^ and a reproducibility of 0.3–0.4 mm^10^ 3D stereophotogrammetry is able to capture small differences between these time‐points. Indeed, the outcomes of the technique lead to changes in the treatment plan of our current daily clinical practice.

*Di Giovanni et al*. stated that cone beam computer tomography (CBCT) is also a reliable technique to assess bony as well as soft tissue changes (including the skin surface).^18^ There is a concern, however, that contraction of the facial muscles interferes with the absolute thickness of facial soft tissue measured with CBCT, potentially leading to a bias in the measured values. Second, one may question the clinical relevance of bony tissue evaluation of a young growing individual since reconstructive and aesthetical surgery is often advised after the face is fully developed. At last, even though it is less than a traditional CT‐scan, CBCT still gives a radiation dose to the young patient.

The main limitations of the presented study are the limited number of included patients and the retrospective design. Since 3D stereophotogrammetry was a new imaging modality for patients with linear scleroderma of the face, 3D photographs of patients were collected at different stages of their disease and treatment phase. Further prospective research with a larger study population, 3D photographs prior to starting systemic treatment and a standardized protocol with a longer follow‐up period is recommended to evaluate its use in monitoring progression of facial asymmetry. The main limitations of the 3D technique for daily clinical practice is the fact that disease activity on the scalp is difficult to monitor. Also, the currently used 3D photogrammetry technique is quite expensive, which makes it challenging to instantly implement it in every hospital. Comparing the outcomes of the currently used camera set‐up with a less expensive handheld camera system would be interesting to investigate in further research in order to reduce costs.

In conclusion, this retrospective case series shows the potential of 3D stereophotogrammetry in patients with scleroderma of the face. This safe, validated and non‐invasive imaging modality is able to detect changes in progression of asymmetry over time and may therefore be a useful tool to help clinicians to objectively monitor progression of linear scleroderma of the face over time in a growing individual.

## AUTHOR CONTRIBUTION

**Rutger ter Horst:** Investigation, Writing – original draft. **Thomas J. J. Maal:** Software, Writing – review & editing. **Martien J. J. de Koning:** Supervision. **Jorre S. Mertens:** Supervision. **Ellen J. H. Schatorjé:** Writing – review & editing. **Esther P. Hoppenreijs:** Writing – review & editing. **Marieke M. B. Seyger:** Supervision, Writing – review & editing.

## CONFLICTS OF INTEREST

None to declare.

## ETHICS STATEMENT

This study was conducted in accordance with the World Medical Association Declaration of Helsinki on medical research ethics. The study was approved by the institutional medical ethical authority (file number W13_303 # 13.17.373) and informed consent was acquired for all patients who were enrolled in the study. All image data were anonymized and de‐identified prior to analysis.

## Data Availability

The data that support the findings of this study are available on request from the corresponding author. The data are not publicly available due to privacy or ethical restrictions.
